# Tooth Retrospective Dosimetry Using Electron Paramagnetic Resonance: Influence of Irradiated Dental Composites

**DOI:** 10.1371/journal.pone.0131913

**Published:** 2015-06-30

**Authors:** Céline M. Desmet, Andrej Djurkin, Ana Maria Dos Santos-Goncalvez, Ruhong Dong, Maciej M. Kmiec, Kyo Kobayashi, Kevin Rychert, Sébastien Beun, Julian G. Leprince, Gaëtane Leloup, Philippe Levêque, Bernard Gallez

**Affiliations:** 1 Biomedical Magnetic Resonance Research group, Louvain Drug Research Institute, Université catholique de Louvain, Brussels, Belgium; 2 School of Dentistry and Stomatology, Université catholique de Louvain, Brussels, Belgium; 3 Advanced Drug Delivery and Biomaterials Research Group, Louvain Drug Research Institute, Université catholique de Louvain, Brussels, Belgium; 4 EPR Center for the Study of Viable Systems, Geisel School of Medicine at Dartmouth, Hanover, NH, United States of America; 5 Center for Research and Engineering on Biomaterials CRIBIO, Université catholique de Louvain, Brussels, Belgium; Northwestern University Feinberg School of Medicine, UNITED STATES

## Abstract

In the aftermath of a major radiological accident, the medical management of overexposed individuals will rely on the determination of the dose of ionizing radiations absorbed by the victims. Because people in the general population do not possess conventional dosimeters, after the fact dose reconstruction methods are needed. Free radicals are induced by radiations in the tooth enamel of victims, in direct proportion to dose, and can be quantified using Electron Paramagnetic Resonance (EPR) spectrometry, a technique that was demonstrated to be very appropriate for mass triage. The presence of dimethacrylate based restorations on teeth can interfere with the dosimetric signal from the enamel, as free radicals could also be induced in the various composites used. The aim of the present study was to screen irradiated composites for a possible radiation-induced EPR signal, to characterize it, and evaluate a possible interference with the dosimetric signal of the enamel. We investigated the most common commercial composites, and experimental compositions, for a possible class effect. The effect of the dose was studied between 10 Gy and 100 Gy using high sensitivity X-band spectrometer. The influence of this radiation-induced signal from the composite on the dosimetric signal of the enamel was also investigated using a clinical L-Band EPR spectrometer, specifically developed in the EPR center at Dartmouth College. In X-band, a radiation-induced signal was observed for high doses (25-100 Gy); it was rapidly decaying, and not detected after only 24h post irradiation. At 10 Gy, the signal was in most cases not measurable in the commercial composites tested, with the exception of 3 composites showing a significant intensity. In L-band study, only one irradiated commercial composite influenced significantly the dosimetric signal of the tooth, with an overestimation about 30%. In conclusion, the presence of the radiation-induced signal from dental composites should not significantly influence the dosimetry for early dose assessment.

## Introduction

The risk of a major radiological incident, resulting from an accident in a nuclear power plant, such as in Fukushima, or from a terrorist device emitting radiation, has drawn increasing attention over the last years. Among the different consequences of such incident, the determination of the amount of the exposure and the appropriate management of overexposed individuals will be a major parameter [1, 2]. Those scenarios are studied and handled by many different actors, both on a national and international level, as official institutions have developed several programs aiming at developing tools to face those situations. To cite only a few examples, the International Atomic Energy Agency (IAEA), through its Incident and Emergency Centre [3], has published various guidelines and technical tools covering preparedness and response to nuclear and radiological emergencies [4, 5]; NATO Science and Technology Organisation also coordinates a task group (Ionizing Radiation Bioeffects and Countermeasures HFM-222) promoting research on biodosimetry [6–8]; at the US level, the National Institute of Allergy and Infectious Diseases (NIAID) coordinates a joint program “Radiation and Nuclear Countermeasures Program” with the US Department of Health and Human Services [9–11]; the Biomedical Advanced Research and Development Authority (BARDA) of the US-HSS is also fostering developments in biodosimetry in case of mass casualty event [9, 10]. Networks are currently also developing at the European level, such as the European Network of Biodosimetry RENEB [12] or EURADOS [13–15].

In this context, a key parameter is the determination of the dose of ionizing radiations absorbed by the overexposed individuals, as this will determine the appropriate medical treatment strategy [2]. The difficulty is that people in the general population do not possess dosimeters such as those available for workers in controlled areas of nuclear facilities. The dose must consequently be evaluated after the fact, using retrospective dosimetry or dose reconstruction methods [16]. Several approaches are available for retrospective dosimetry, namely cytogenetic assays, changes in genes and gene products, changes in metabolites, mathematical modelling using MonteCarlo simulation, and biophysical methods [17]. In the hypothesis of a large scale incident, there will be an immediate and urgent need to perform triage of a large number of people at risk. This will rely on a first assessment of the individual dose, which should preferably occur in the field and very quickly. Progress has been made in many biodosimetry approaches [6, 18], and it has been suggested that Electron Paramagnetic Resonance (EPR) biodosimetry, using tooth enamel of victims as a natural dosimeter, could help in the early assessment of the dose for initial triage. Indeed, ionizing radiations generate very stable carbonate free radicals within the hydroxyapatite matrix of the enamel, in a linear dose-dependent manner, and can be readily detected with EPR [19]. Over the last years, the Dartmouth Center for Medical Countermeasures Against Radiation (CMCR) has developed specific low frequency (L-band) EPR spectrometers for non invasive in vivo measurements, directly in the subject’s mouth. Importantly, those spectrometers are in the field deployable, and particularly adapted for mass triage. They are currently under extensive evaluation [20–24].

**Fig 1:**
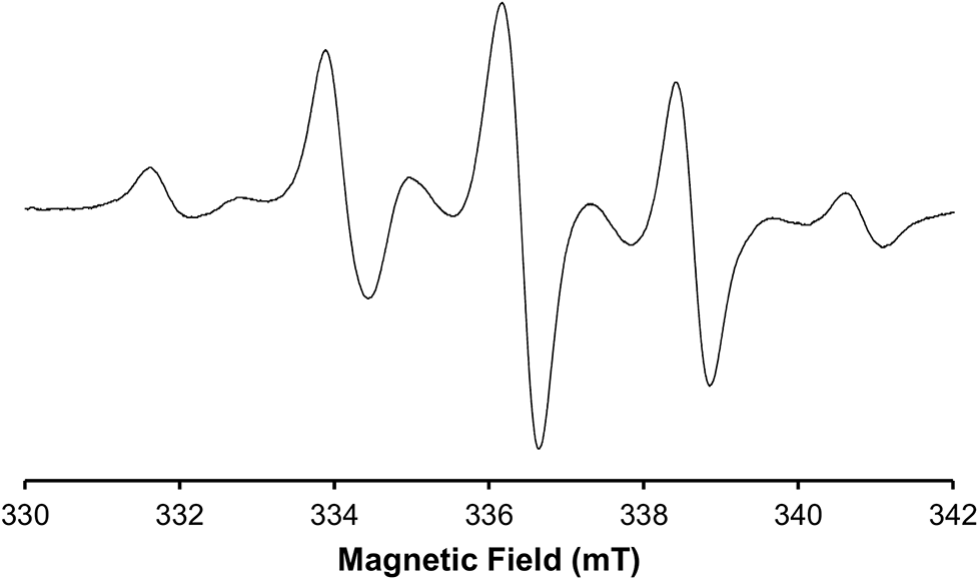
Typical polymerization signal recorded in a commercial composite (Filtek Supreme Ultra).

Apart from the issues linked to the instrumentation, factors affecting the dosimeter itself, and consequently also potentially affecting the dosimetric signal, should be investigated in order to fully validate the method. In a previous work, we investigated the influence of tooth restorations on the dosimetric signal, and more specifically the influence of the signal due to the photopolymerization process [25]. Indeed, dimethacrylate based composites used for restoration of teeth are photopolymerized in situ using visible light, and this polymerization process generates stable free radicals giving a strong EPR signal [26, 27]. Free radicals created in the organic matrix will recombine at the end of the polymerization process, but as the vitrification progresses, the mobility of the free radicals becomes too low to allow recombination, and they remain trapped in the polymerized matrix [28], giving an intense and complex 9 lines spectrum [27]. This signal was shown to fade more or less rapidly, depending on the storage environment [29], likely due to a hydroperoxidation phenomenon [30]. We observed that a strong polymerization signal, the complex 9 lines spectrum, was indeed systematically present in every composite tested (Fig 1), with the same resonance frequency as the dosimetric signal of the enamel. This “polymerization signal” was nevertheless rapidly decaying with time so that, in L-band, it was hardly detectable 18 days after the initiation of the polymerization, and its influence on the dosimetric signal could be neglected [25]. After decaying of this polymerization signal, in some composites, a stable atypical signal, resembling to an immobilized nitroxide signal, was also detected.

Restorations are not easily detected by untrained operators, but could be with the help of an appropriate device such as a UV lamp. One strategy could consist in excluding restored teeth from measurements, and consider measuring an adjacent tooth, although it is not granted that they would not bear any restoration.

So the systematic exclusion of restored teeth would significantly impact the global efficacy of the technique. On the contrary, a procedure designed to include the measurement of restored teeth, including a correction of the signal if necessary, would offer a better adaptability of the technique. In this context, in the present study, we raised the issue of another possible EPR signal, different from the photopolymerization signal, induced in the composite when it is exposed to ionizing radiations, a situation that would be very likely for victims of a radiological incident, as teeth of a large part of the population do bear restorations. Dental composites contain a large fraction of inorganic fillers (circa 50 to 80% wt), such as silica particles or barium glasses [31], materials which are known to give an EPR signal when exposed to ionizing radiations [32–34]. Free radicals are also likely to be induced in the organic matrix itself, where they can remain stable for an undetermined period of time. The aim of the present study was to screen irradiated composites for a possible radiation-induced EPR signal, to characterize it in terms of intensity and stability, and evaluate the possible interference with the dosimetric signal of the tooth enamel. We investigated the most common commercial composites available on the market, and experimental compositions for a possible class effect.

In the first part of this study, the effect of the radiation at high dose (100 Gy) was investigated using a X-band spectrometer, because it allowed higher sensitivity. The signal was characterized and its stability investigated by means of kinetics study. The delivered dose was progressively lowered to 10 Gy in order to determine the level of dose at which no radiation-induced signal could be observed.

In a second part of the study, the influence of the radiation-induced signal from the composite on the dosimetric signal of the enamel was also investigated using a clinical L-Band EPR spectrometer, developed in the EPR center at the Dartmouth College and designed for in vivo measurements in humans, and an experimental setting close to a realistic situation.

## Materials and Methods

This study was divided in two parts. First, we investigated the occurrence of a radiation-induced signal in the resins and composites using X-band EPR spectroscopy, because X-band allowed a higher sensitivity, a better characterization of the signal, and faster measurements. Measurements were performed on small bars of composites submitted to various doses of radiation, first at high dose (100 Gy) in order to characterize the signal and study its stability, then at lower doses in order to determine whether a threshold was observable.

In a second part, we checked the influence of the irradiated composite on the dosimetric signal of the tooth, using a setting mimicking more realistic conditions. Bars of composites were irradiated in the low dose range and inserted in a tooth, irradiated separately at the same dose. Measurements were carried out using a L-band spectrometer, developed in Dartmouth (USA) specifically for in the field direct measurement of teeth in the mouth of individuals.

### Part I: characterization of the radiation-induced signal in restorative material using X-band

#### Composites

Nineteen composites were selected among the commercial composites the most widely used by dentists and routinely used for restoration of incisors (Table 1). These composites were identical to those characterized in our previous work about the EPR signal of restorative materials [25].

**Table 1:** Commercial composites selected for this study among the most widely used on the market.

Commercial composites	Shade	Lot	Brand
Filtek Supreme Ultra	A3	N265426	3M-ESPE, St Paul, MN, USA
Venus Diamond	A3	010040	Heraeus-Kulzer, Wehrheim, Germany
IPS Empress Direct	A3	P02374	Ivoclar-Vivadent, Schaan, Liechtenstein
Tetric EvoCeram	A3	P11989	Ivoclar-Vivadent, Schaan, Liechtenstein
Amaris	O3	1121316	Voco GmbH, Cuxhaven, Germany
GrandioSo	A3	1120117	Voco GmbH, Cuxhaven, Germany
Gradia Direct X	A3	1103081	GC Europe N.V., Leuven, Belgium
GC Kalore	A3	1007201	GC Europe N.V., Leuven, Belgium
Ice	A3	110150T	Southern Dental Industries, Australia
N’Durance	A3	11011OB	Septodont, Saint-Maur-des-Fossés, France
Clearfil AP-X	A3	1383AA	Kuraray Europe GmbH, Hattersheim am Main, Germany
Clearfil Majesty Esthetic	A3	0038CA	Kuraray Europe GmbH, Hattersheim am Main, Germany
Synergy D6	A3	C42276	Coltène-Whaledent, Langenau, Germany
Esthet-X HD	A3	1106102	Dentsply Caulk, Milford, DE, USA
TPH3	A3	1110000495	Dentsply Caulk, Milford, DE, USA
Ceram-X	A3	1110000028	Dentsply Caulk, Milford, DE, USA
Artiste Nano	A3	3666316	Pentron Clinical, Orange, CA, USA
Simile	A3	4328025	Pentron Clinical, Orange, CA, USA
Herculite Ultra	A3	3978906	Kerr Corporation, Orange, CA, USA

#### Experimental resins

Experimental resins were prepared using different proportions of the most common monomers. Bisphenol A glycidyl dimethacrylate (Bis-GMA), triethylene glycol dimethacrylate (TEGDMA), urethane dimethacrylate (UDMA), ethoxylated bisphenol A glycidyl dimethacrylate (Bis-EMA-2 and Bis-EMA-15) were all purchased from Sigma-Aldrich (Belgium). Fourteen compositions of these monomers were prepared according to Sideridou et al. (Table 2) [35]. Each composition contained 2% (molar) camphorquinone (Sigma-Aldrich, Belgium) as the photo-initiator and 2% (molar) ethyl-4-dimethylaminobenzoate (Sigma-Aldrich, Belgium) as the co-initiator.

**Table 2:** Composition of experimental resins.

Monomers	Molar ratio	%weight
G	1	100
T	1	100
U	1	100
E-15	1	100
E-2	1	100
G/T	0.3582/0.6418	50/50
G/T	0.5659/0.4341	70/30
G/U	0.3582/0.6418	37.8/62.2
G/U	0.5659/0.4341	58.7/41.3
G/E-15	0.3582/0.6418	43.2/56.8
G/E-15	0.5659/0.4341	64/36
G/T/U/E-15	0.5660/0.2340/0.1/ 0.1	65.7/15.2/10.6/8.5
G/T/U/E-15	0.5660/0.1340/0.15/0.15	63.7/8.4/15.5/12.4
G/U/E-15	0.5660/0.2170/ 0.2170	61.3/21.5/17.2

G: Bis-GMA, T: TEGDMA, U: UDMA, E-15: Bis-EMA (15-ethoxy/phenol), E-2: Bis-EMA (2-ethoxy/phenol).

#### Polymerization of samples

Composites and resins were polymerized as already described [25]. Briefly, 30 mg of resin material was cast in a PTFE mould (7 mm x 1.4 mm x 1.4 mm) and polymerized using a BluePhase G2 lamp (Ivoclar Vivadent, Schaan, Liechtenstein) at high power (1200 mW/cm^2^) during 20 seconds. Samples were stored in dry conditions and in the dark during all the study.

All resins were used at least six months after polymerization in order to ensure a total decay of the photopolymerization signal.

#### Irradiation of samples–Occurrence of the radiation-induced signal in composites and resins

In order to detect the occurrence of a possible radiation-induced signal, commercial composites and experimental resins were first all irradiated at the dose of 100 Gy (dose absorbed in water) with a ^137^Cs gamma irradiator (IBL637, Oris Industrie), in order to detect and characterize a possible radiation-induced signal. Other samples were then irradiated at lower doses (50, 25 and 10 Gy) to determine the level of dose not giving any signal. All samples were polymerized at least six months before the external irradiation.

#### EPR X-band measurements

X-band measurements were carried out with a Miniscope MS200 spectrometer (Magnettech, Berlin, Germany) operating at ~9.5 GHz. The spectrometer was calibrated with a standard of 2,2-diphenyl-1-picrylhydrazyl (dpph) (batch # 1204D191 Bruker Biospin, Rheinstetten, Germany) before and after the measurements of samples. The acquisition parameters were as follows: center field: 335.600 mT, sweep field: 13.000 mT, acquisition time: 30 s, number of points: 4096, number of scans: 1, modulation frequency: 100 kHz, modulation amplitude: 0.1 mT, power: 0.5 mW, gain: 300. The signal intensity was measured as the peak-to-peak height of the central peak of the spectrum.

As the measurements had to be carried out over several hours, and repeated on several days, the signal intensity was normalized with the signal intensity of the dpph standard and expressed as normalized units (n.u.). This ensured quantitative data by minimizing any drift of the signal and intraday as well as interday variations.

#### Kinetics of decay of the radiation-induced signal in composites and resins

The study of the decay kinetics of the radiation-induced signal was performed on composites and resins at least six months after their initial polymerization, after the decay of the polymerization signal. For this part of the study, samples still presenting an atypical residual (nitroxide-like) signal were excluded, as a low radiation-induced signal would be masked by the residual signal and would prevent any further measurements. The delivered dose was 100 Gy. Measurements were carried out in X-band for sensitivity reasons, in triplicate, and were started one hour after the end of the irradiation. The EPR signal was then measured once a day during one week, and once a week during one month when necessary. Decay curves were fitted with a monoexponential decay model using Prism 5 (GraphPad Software, La Jolla, CA, USA) where applicable.

#### Powder of dental enamel

A powder of dental enamel (164 mg) was obtained by crushing the crown of a molar tooth after removal of the dentin. The molar was obtained from the collection of the laboratory of anatomy at the faculty of medicine, Université catholique de Louvain. The powder was irradiated at 5 and 10 Gy with the same ^137^Cs gamma irradiator. The ratio of the mass of composite to the mass of enamel was 0.18, which is considered as representative of a medium sized restoration.

### Part II: Influence of the radiation-induced signal on the dosimetric signal using clinical L-band

#### Influence of irradiated restoration on the dosimetric signal from an irradiated tooth

A cavity of 2.1 mm was prepared in an incisor obtained from a cadaver (anatomy department, Université catholique de Louvain). The tooth was then submitted to a dose of 15 Gy (dose to water) using a ^137^Cs gamma irradiator at a dose rate of 1Gy/min (JL Shepherd and Associates, San Fernando, CA, USA). The EPR dosimetric signal was measured using a L-band spectrometer operating at 1.15 GHz and an associated magnetic field of 41 mT [20–24]. The parameters of acquisition were the following: modulation amplitude 0.4 mT, modulation frequency 21780 Hz, sweep width 4.0 mT, 1024 points/scan, scan time 3.0 s, time constant 5 ms, power 6 dB (25 mW). Twenty consecutive scans were performed for each sample. Small bars (7 mm x 1.4 mm x 1.4 mm) of commercial composites, polymerized at least six months earlier, were exposed to the same 15 Gy dose. After irradiation, the bar was inserted in the cavity prepared in the tooth, and the tooth was measured a second time in order to check the possible influence of the irradiated composite on the dosimetric signal, using the same parameters. Measurements were performed 5 hours after the irradiation. This time point was selected so that the measurements would be carried out in a timeframe as close as possible from the initial irradiation, when the signal was suspected to be the most intense and consequently with the strongest influence on the dosimetric signal. Five acquisitions were performed for each composite. Means were compared using student t-test.

#### Ethics statement

Human samples were obtained and used after approval of the study by the local ethics committee “Commission d’éthique biomédicale hospitalo-facultaire” from the university hospital “Cliniques universitaires Saint-Luc” (IRB 00001530).

Teeth were obtained from people who offered their body for medical teaching and research. Samples were anonymized prior to analysis.

## Results

### Radiation-induced signal in commercial composites

**Fig 2:**
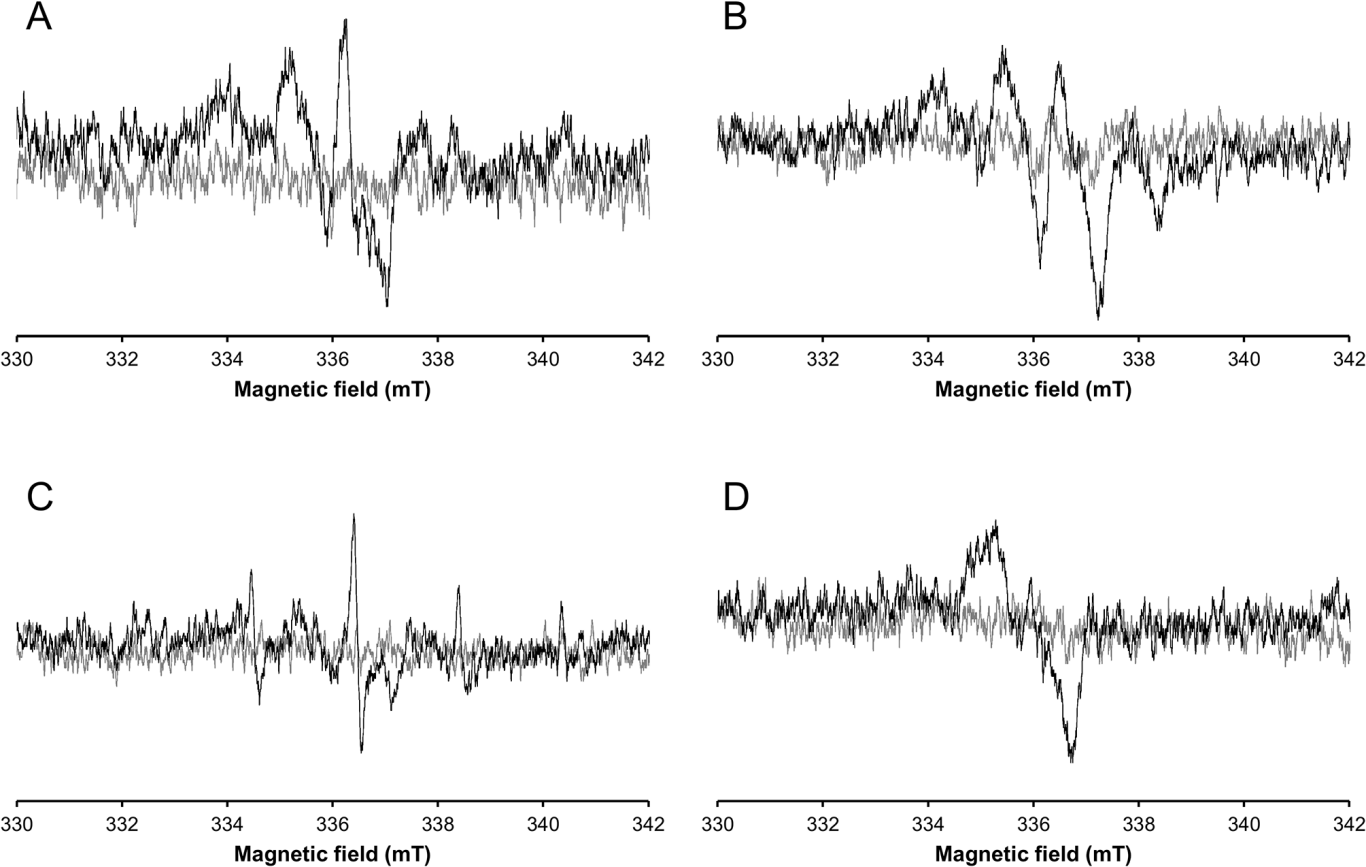
Illustrative examples of some radiation-induced signals recorded in commercial composites before irradiation (gray line) and irradiated at 100 Gy (black line). Filtek Supreme Ultra (A), Venus Diamond (B), N’Durance (C), Clearfil Majesty Esthetic (D).

After a 100 Gy irradiation, a radiation-induced EPR signal was clearly observed in X-band in 14 composites. This radiation-induced signal is characterized by various and complex spectral shapes (Fig 2). Normalized intensities (n.u.) ranged from 0.37 n.u. to 0.82 n.u. (Table 3), one hour after irradiation. After a 10 Gy irradiation, the radiation-induced signal was observable in only 3 composites (N’Durance, Herculite Ultra, Clearfil Majesty Esthetic). No signal was observed in the 11 other commercial composites. At 10 Gy, the radiation-induced signal in commercial composites ranged from 0.29 n.u. to 0.33 n.u. whereas the EPR dosimetric signal measured in irradiated enamel had an intensity of 0.73 n.u. at 10 Gy and 0.42 n.u. at 5 Gy.

**Table 3:** Normalized intensities of EPR signal recorded in commercial composites irradiated at 10, 25, 50 and 100 Gy in X-band.

Commercial composites	Signal intensity at 10 Gy (n.u.)	Signal intensity at 25 Gy (n.u.)	Signal intensity at 50 Gy (n.u.)	Signal intensity at 100 Gy (n.u.)
Filtek Supreme Ultra	n.d.	0.42	0.53	0.76
Venus Diamond	n.d.	0.55	0.74	0.82
IPS Empress Direct	Nitroxide-like	Nitroxide-like	Nitroxide-like	Nitroxide-like
Tetric EvoCeram	Nitroxide-like	Nitroxide-like	Nitroxide-like	Nitroxide-like
Amaris	n.d.	0.35	0.37	0.43
GrandioSo	Nitroxide-like	Nitroxide-like	Nitroxide-like	Nitroxide-like
Gradia Direct X	n.d.	0.41	0.38	0.40
GC Kalore	n.d.	0.30	0.31	0.41
Ice	n.d.	0.35	0.37	0.42
N’Durance	0.29	0.34	0.44	0.80
Clearfil AP-X	Nitroxide-like	Nitroxide-like	Nitroxide-like	Nitroxide-like
Clearfil Majesty Esthetic	0.30	0.46	0.60	0.70
Synergy D6	n.d.	0.35	0.35	0.42
Esthet-X HD	n.d.	0.34	0.36	0.56
TPH3	n.d.	0.44	0.45	0.60
Ceram-X	Nitroxide-like	Nitroxide-like	Nitroxide-like	Nitroxide-like
Artiste Nano	n.d.	0.34	0.31	0.37
Simile	n.d.	0.33	0.38	0.47
Herculite Ultra	0.33	0.40	0.41	0.46

Units are normalized to the dpph signal intensity (n.u.).

n.d.: not detectable.

**Fig 3:**
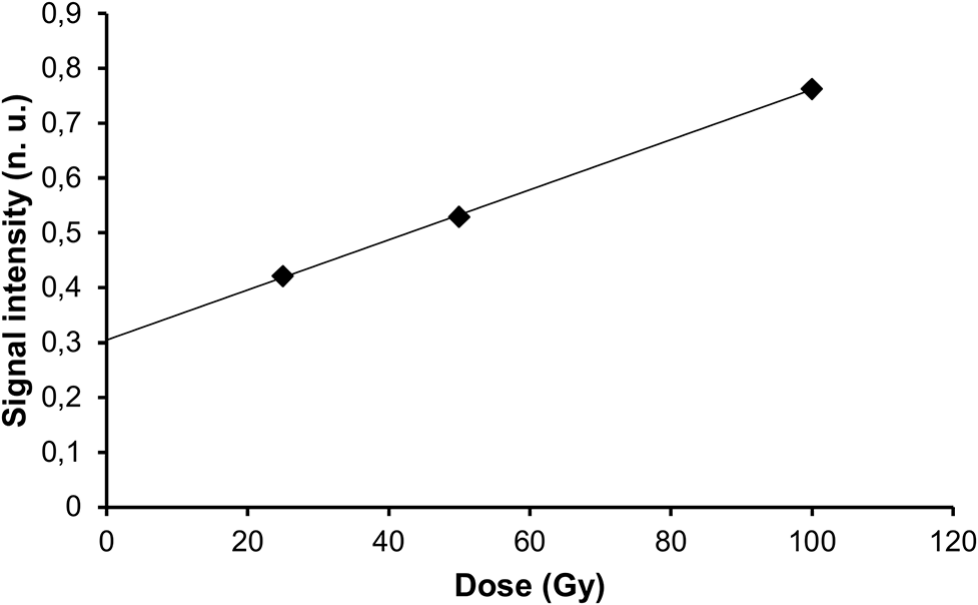
Relationship between radiation-induced signal intensity and irradiation dose in the commercial composite Filtek Supreme Ultra.

Above 10 Gy, the relation between the dose and the signal intensity was compatible with a linear model in 8 of the 14 composites (r^2^ > 0.90), in the interval of doses tested (Fig 3 and Table 4). Below 10 Gy, no signal was observed (S1 Fig), except noise around 0.13 n.u., so that the value of the intercept should not be used to extrapolate the intensity of the radiation-induced signal at lower doses, nor anticipate interference with the dosimetric signal in that particular range of doses.

**Table 4:** Signal-dose regression in commercial composites. Parameters of linear regression model.

Commercial composites	Equation	R^2^
Filtek Supreme Ultra	y = 0.0046x + 0.30	1.00
Venus Diamond	y = 0.0033x + 0.51	0.84
IPS Empress Direct	n.a.	
Tetric EvoCeram	n.a.	
Amaris	y = 0.0011x + 0.32	0.99
GrandioSo	n.a.	
Gradia Direct X	y = -0.0001x + 0.40	0.02
GC Kalore	y = 0.0015x + 0.26	0.93
Ice	y = 0.0009x + 0.33	1.00
N’Durance	y = 0.0057x + 0.20	0.98
Clearfil AP-X	n.a.	
Clearfil Majesty Esthetic	y = 0.0041x + 0.32	0.87
Synergy D6	y = 0.0010x + 0.32	0.89
Esthet-X HD	y = 0.0031x + 0.24	0.94
TPH3	y = 0.0023x + 0.36	0.93
Ceram-X	n.a.	
Artiste Nano	y = 0.0005x + 0.31	0.43
Simile	y = 0.0019x + 0.28	1.00
Herculite Ultra	y = 0.0012x + 0.35	0.80

n.a.: not applicable, nitroxide-like signal.

**Fig 4:**
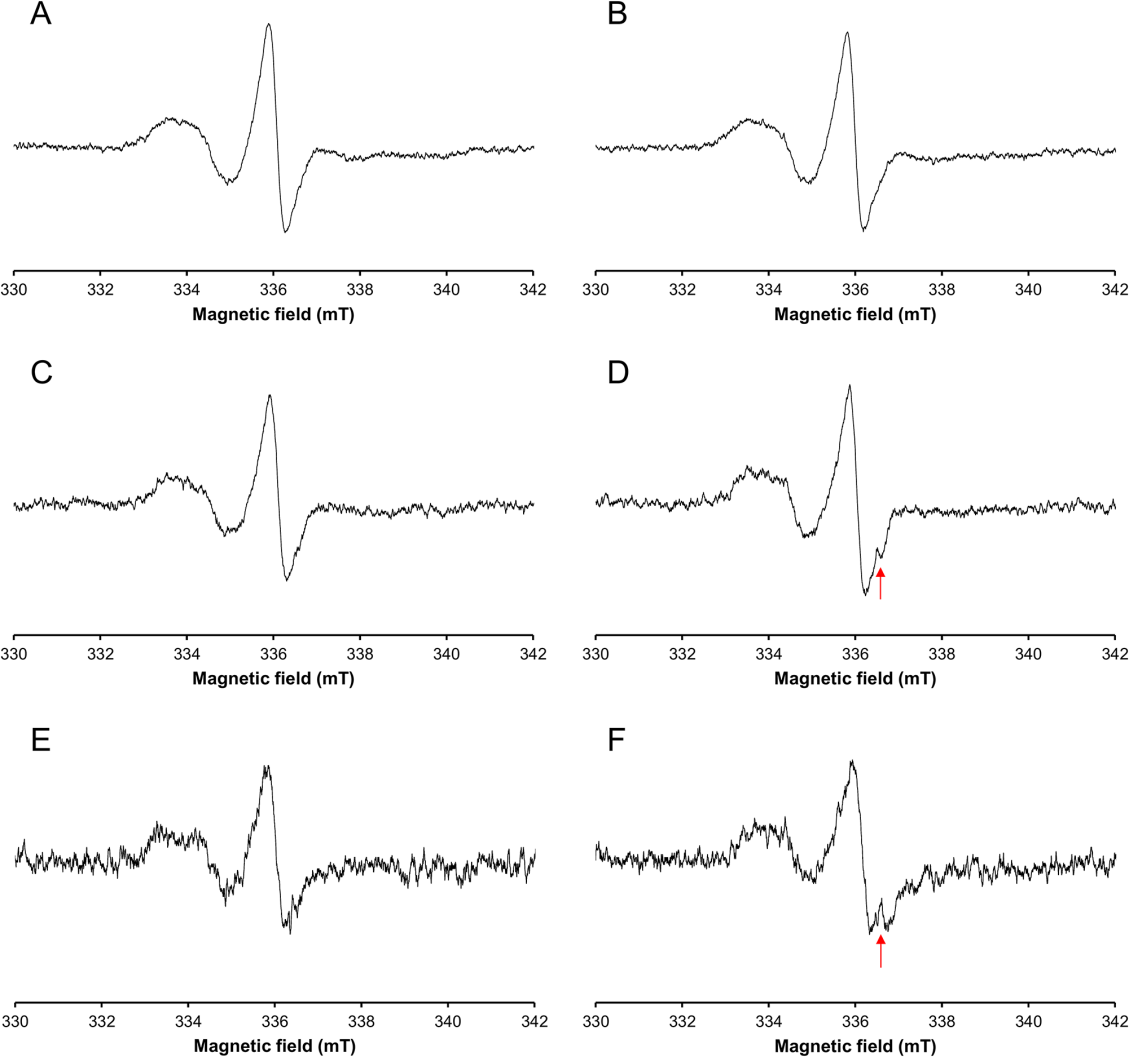
Nitroxide-like signal recorded in GrandioSo (A-B), IPS Empress Direct (C-D) and Tetric EvoCeram (E-F) before (A-C-E) and after irradiation (100 Gy) (B-D-F). Arrow shows the contribution of the radiation-induced signal.

Among the five other composites, showing a residual stable nitroxide-like signal at the onset of the irradiation, no particular additional signal was observed for GrandioSo, Clearfil AP-X & Ceram-X, even after a 100 Gy irradiation. However, for IPS Empress Direct and Tetric EvoCeram a small extra contribution was noted, superimposed on the nitroxide-like signal (Table 3 and Fig 4). This signal was not detected after irradiation at lower doses.

### Radiation-induced signal in experimental resins

In the pure monomers, after a 100 Gy irradiation, no radiation-induced signal was measured in the TEGDMA nor Bis-EMA-15 monomers. In the UDMA, Bis-GMA and Bis-EMA-2 monomers, a signal was observed with an intensity ranging from 0.57 n.u. to 1.30 n.u. (Table 5). In some experimental compositions of monomers irradiated at 100 Gy, an intense radiation-induced signal was detected (Table 5). A class effect was observed in experimental resins: a radiation-induced signal was detected in the G/T, G/U and G/(T)/U/E-15 compositions. No radiation-induced signal was detected in G/E-15 compositions.

**Table 5:** Normalized intensities of EPR signal recorded in experimental resins irradiated at 10, 25, 50 and 100 Gy in X-band.

Experimental resins	Signal intensity at 10 Gy (n.u.)	Signal intensity at 25 Gy (n.u.)	Signal intensity at 50 Gy (n.u.)	Signal intensity at 100 Gy (n.u.)
G 100%	0.68	0.87	1.14	1.30
T 100%	n.d.	n.d.	n.d.	n.d.
U 100%	0.52	0.66	0.79	0.97
E-15 100%	n.d.	n.d.	n.d.	n.d.
E-2 100%	n.d.	0.37	0.44	0.57
G/T 0.3582/0.6418	0.71	0.75	1.03	1.31
G/T 0.5659/0.4341	0.52	0.75	0.93	1.17
G/U 0.3582/0.6418	0.66	0.66	0.95	1.11
G/U 0.5659/0.4341	0.61	0.75	0.79	1.17
G/E-15 0.3582/0.6418	n.d.	n.d.	n.d.	n.d.
G/E-15 0.5659/0.4341	n.d.	n.d.	n.d.	n.d.
G/T/U/E-15 0.5660/0.2340/0.1/0.1	0.43	0.44	0.58	0.76
G/T/U/E-15 0.5660/0.1340/0.15/0.15	0.37	0.35	0.40	0.57
G/U/E-15 0.5660/0.2170/0.2170	0.36	0.36	0.48	0.66

Units are normalized to the dpph signal intensity (n.u.).

G: Bis-GMA, T: TEGDMA, U: UDMA, E-15: Bis-EMA (15-ethoxy/phenol), E-2: Bis-EMA (2-ethoxy/phenol).

n.d.: not detectable.

**Fig 5:**
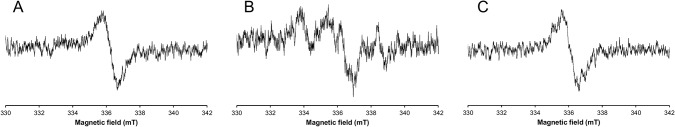
Radiation-induced signal recorded in irradiated experimental resins at 100 Gy. Bis-GMA 100% (A), UDMA 100% (B), G/T 0.3582/0.6418 (C).

The shape of the radiation-induced signal, a single broad line spectrum (linewidth = ~1 mT, gvalue = 2.0039), different from the complex radiation-induced signal observed in commercial composites, was the same for all samples with the exception of UDMA monomer showing a more complex signal with an overall linewidth of 5 mT (Fig 5).

**Fig 6:**
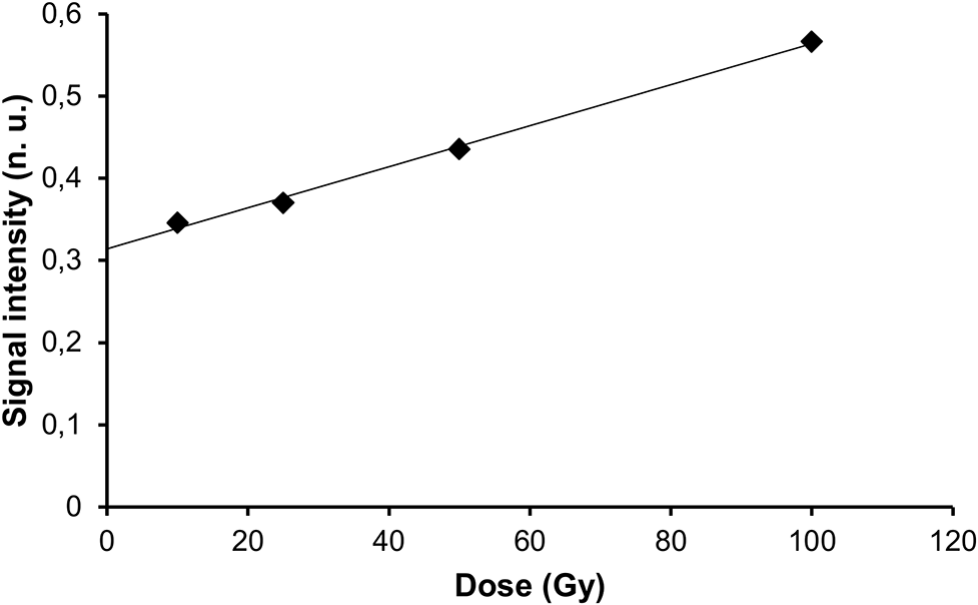
Relationship between radiation-induced signal intensity and irradiation dose in experimental resin Bis-EMA-2 (E-2).

When present, the radiation-induced signal increased linearly with the dose (Fig 6 and Table 6).

**Table 6:** Signal-dose regression in experimental resins. Parameters of linear regression model.

Experimental resins	Equation	R^2^
G 100%	y = 0.0067x + 0.69	0.90
T 100%	n.a.	
U 100%	y = 0.0048x + 0.51	0.96
E-15 100%	n.a.	
E-2 100%	y = 0.0025x + 0.31	0.99
G/T 0.3582/0.6418	y = 0.0069x + 0.63	0.97
G/T 0.5659/0.4341	y = 0.0068x + 0.53	0.94
G/U 0.3582/0.6418	y = 0.0054x + 0.60	0.92
G/U 0.5659/0.4341	y = 0.0060x + 0.55	0.96
G/E-15 0.3582/0.6418	n.a.	
G/E-15 0.5659/0.4341	n.a.	
G/T/U/E-15 0.5660/0.2340/0.1/0.1	y = 0.0049x + 0.29	0.96
G/T/U/E-15 0.5660/0.1340/0.15/0.15	y = 0.0023x + 0.32	0.86
G/U/E-15 0.5660/0.2170/0.2170	y = 0.0035x + 0.31	0.97

n.a.: not applicable

G: Bis-GMA, T: TEGDMA, U: UDMA, E-15: Bis-EMA (15-ethoxy/phenol), E-2: Bis-EMA (2-ethoxy/phenol).

### Decay kinetics of the radiation-induced signal in commercial composites

**Fig 7:**
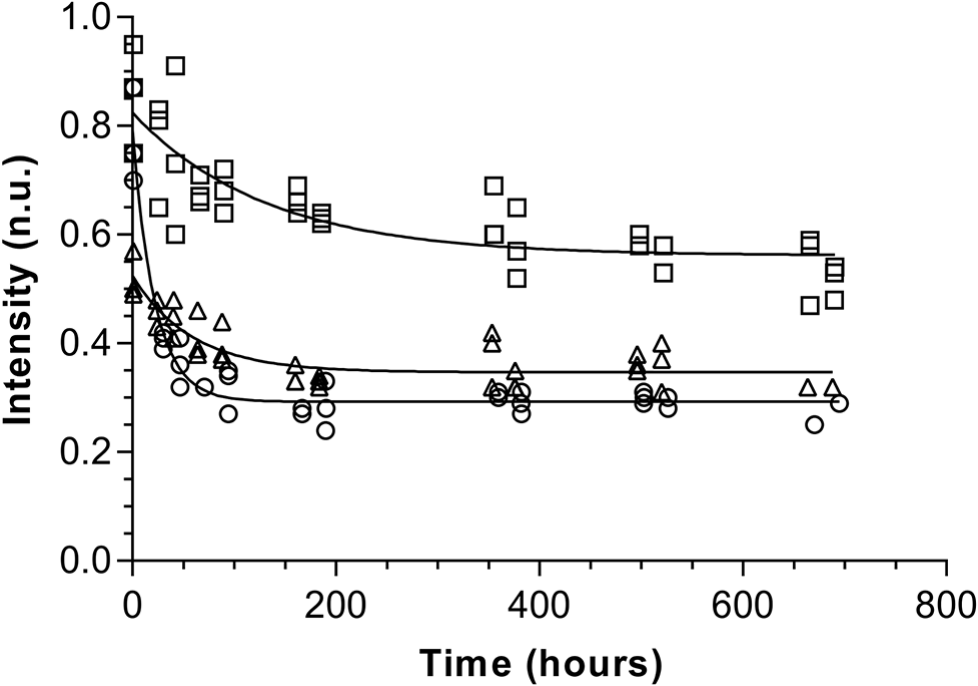
Monoexponential fitting of the decay curves of the radiation-induced signal intensity in composites. Filtek Supreme Ultra ○, N’Durance ☐, TPH3 △.

The decay kinetics study, performed over a one month period, showed that the radiation-induced signal in commercial composites was unstable. In 7 out of the 14 composites selected for the decay kinetics, no signal was observed after only 24 hours post irradiation (Table 7). A slower decay was observed for the 7 other composites, compatible with a monoexponential decay with t_1/2_ ranging from 15 to 91 hours (Table 7 and Fig 7). The signal completely disappeared in one week, except for the N’Durance composite, showing a more stable signal, still present one month after the initial irradiation.

**Table 7:** Decay kinetics parameters for commercial composites using a monoexponential model.

Commercial composites	t_1/2_ (h) (CI 95%)
N’Durance	91 (55–262)
Gradia Direct X	60 (44–96)
TPH3	41 (28–78)
Clearfil Majesty Esthetic	29 (24–38)
Venus Diamond	25 (20–34)
GC Kalore	24 (16–53)
Filtek Supreme Ultra	15 (13–19)
Amaris	n.a.
Ice	n.a.
Synergy D6	n.a.
Esthet-X HD	n.a.
Artiste Nano	n.a.
Simile	n.a.
Herculite Ultra	n.a.
IPS Empress Direct	Nitroxide-like signal
Tetric EvoCeram	Nitroxide-like signal
GrandioSo	Nitroxide-like signal
Clearfil AP-X	Nitroxide-like signal
Ceram-X	Nitroxide-like signal

Half-lifes (T_1/2_) expressed in hours (h) with confidence interval at 95% (CI 95%).

n.a.: not applicable, complete decay < 24h.

### Decay kinetics of the radiation-induced signal in experimental resin

In the experimental resins giving an EPR signal after irradiation, the decay kinetics of the radiation-induced signal was generally fast, with a half-life below 10 hours. UDMA was an exception with a t_1/2_ approaching 100 hours (Table 8).

**Table 8:** Decay kinetics parameters for experimental resins using a monoexponential model.

Experimental resins	t_1/2_ (h) (CI 95%)
G 100%	4.5 (2.9–10.3)
T 100%	-
U 100%	91 (54–292)
E-15 100%	-
E-2 100%	n.d.
G/T 0.3582/0.6418	7.8 (5.0–17.8)
G/T 0.5659/0.4341	13 (8–31)
G/U 0.3582/0.6418	n.d.
G/U 0.5659/0.4341	3.0 (2.2–4.4)
G/E-15 0.3582/0.6418	-
G/E-15 0.5659/0.4341	-
G/T/U/E-15 0.5660/0.2340/0.1/0.1	n.a.
G/T/U/E-15 0.5660/0.1340/0.15/0.15	n.a.
G/U/E-15 0.5660/0.2170/0.2170	n.a.

Half-lifes (t_1/2_) expressed in hours (h) with confidence interval at 95% (CI 95%).

G: Bis-GMA, T: TEGDMA, U: UDMA, E-15: Bis-EMA (15-ethoxy/phenol), E-2: Bis-EMA (2-ethoxy/phenol).

-: no radiation-induced signal. n.a.: not applicable, complete decay < 24h. n.d.: not determined.

### Influence of irradiated composites on the dosimetric signal in tooth

**Fig 8:**
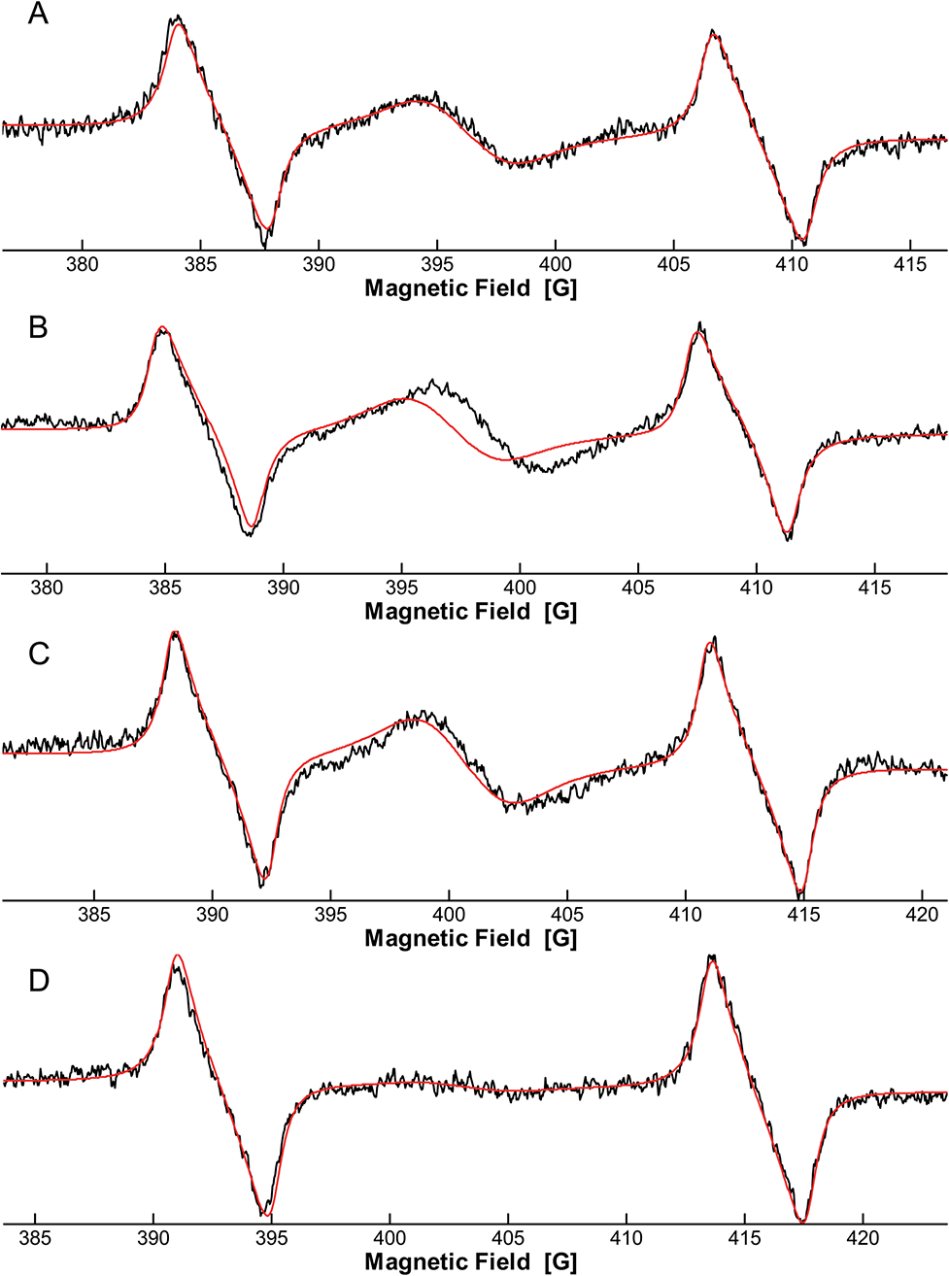
L-band measurement of an irradiated tooth (15 Gy, A), irradiated composite alone (15 Gy, B), irradiated tooth including the irradiated composite (15 Gy, C) and non irradiated tooth (D). Black line is the median of 20 scans. Red line is the fit. Peaks at low and high field are due to the standard used (^15^N-PDT).

Measurements were also performed using the clinical L-band spectrometer developed by the Dartmouth EPR Center for in the field and in vivo measurement of teeth. It was equipped with a resonator specifically designed and optimized for teeth [20]. After irradiation at 15 Gy, the mean dosimetric signal intensity from the enamel of an incisor was 0.30 ± 0.02 a.u. As a cavity was prepared in the tooth before irradiation, it was possible to insert a small bar of commercial composite separately irradiated at the same level of dose (15 Gy), in order to mimic a standard restoration. A second measurement was then performed 5 hours after the irradiation of the composite to check its influence on the dosimetric signal from the enamel. Data for each composite are presented in Table 9. Overall, the signal measured after inclusion of the irradiated bar was very close to the one observed without the restoration (Table 9). A statistically significant difference was observed for only one composite, IPS Empress (Fig 8). A possible influence can not be completely ruled out for GC Kalore, with a p value very close to the significant threshold.

**Table 9:** Intensity of the dosimetric signal in presence of an irradiated restoration (15 Gy).

Commercial composites	Mean (a.u.)	SD	p
Irradiated tooth (15Gy)	0.30	0.02	
Filtek Supreme Ultra	0.32	0.02	0.190
Venus Diamond	0.29	0.03	0.337
**IPS Empress Direct**	**0.39**	**0.03**	**0.001**
Ceram-X	0.33	0.03	0.109
Amaris	0.31	0.03	0.831
GrandioSo	0.33	0.03	0.191
Gradia Direct X	0.31	0.05	0.889
GC Kalore	0.27	0.01	0.014
Ice	0.26	0.03	0.060
N’Durance	0.28	0.03	0.210
Clearfil AP-X	0.29	0.03	0.617
Clearfil Majesty Esthetic	0.29	0.03	0.552
Synergy D6	0.26	0.03	0.047
Esthet-X HD	0.29	0.02	0.331
TPH3	0.27	0.03	0.059
Artiste Nano	0.30	0.03	0.771
Simile	0.28	0.04	0.318
Herculite Ultra	0.33	0.03	0.147

Mean ± SD expressed in arbitrary units (a.u.). P value are significative < 0.01

## Discussion

Retrospective dosimetry using EPR relies on the detection of stable free radicals generated by ionizing radiations in the enamel of teeth. Parameters interfering with this dosimetric signal can potentially affect the accuracy of the dosimetry. Among those factors, the presence of restorations in teeth is a possible concern. In a previous work, we investigated a first aspect which was the occurrence of an EPR signal arising from the photopolymerization of the composite [25]. We concluded that this polymerization signal was quite unstable, and that in most cases only recent restorations would affect the dosimetric signal, because the polymerization signal was not anymore detectable 6 months after the polymerization. Nevertheless, the effect of ionizing radiations on the composites themselves, after their polymerization, had not been investigated, and was an important parameter to consider for further validation of this technology. The purpose of the present study was to evaluate whether the irradiation of restorative material (composites) would induce free radicals in the matrix, either organic or mineral part, and consequently a detectable EPR signal, different from the photopolymerization signal, and also possibly interfering with the dosimetric signal of the enamel.

We investigated the occurrence of an EPR signal after irradiation of polymerized commercial composites and experimental resins, and characterized the signal in terms of shape, intensity and decay kinetics with X-band studies. A radiation-induced signal was observed for very high doses (25–100 Gy), and was generally rapidly decaying. Based on scenarios published by different official authorities dealing with radiation, such as NCRP and other consensus conferences [36–38], this range of doses is only expected in the near vicinity of the nuclear device, and a limited number of individuals would be exposed to doses above 10 Gy. Those victims would present very rapidly severe deterministic effects (vomiting, erythema, neurological syndrome etc.) so that dose evaluation could be based on the symptoms alone, as they would be indicative of a fatal issue on a very short term. Only palliative care would be required in this situation.

At the dose of 10 Gy, the signal was in most cases not measurable in the commercial composites tested. In 3 composites however (N’Durance, Clearfil Majesty Esthetic, Herculite Ultra), a signal with a significant intensity was observed, ranging from 0.29 to 0.33 n.u., compared to the dosimetric signal of enamel for the same delivered dose (0.73 n.u.). The decay of the signal for Herculite Ultra was extremely fast, so that it was not detectable after a few hours. No real influence of this composite is to be expected. The half-life for Clearfil Majesty Esthetic was somewhat longer (29 h), so that a possible influence might be anticipated during the first 24 hours after irradiation. As the t_1/2_ of N’Durance was quite longer (91 h) an overestimation of the dose is to be anticipated. Those results were obtained using high frequency X-band spectrometry to ensure high sensitivity, and a geometry of cavity allowing the detection of the signal from the whole volume of the composite. This must be considered as a worst-case situation. Indeed, in the field, measurements will be carried out using a low frequency L-band spectrometer equipped with a surface resonator, optimized for the detection of the surface enamel of the tooth. The geometrical factor will then be much more favourable to the detection of the enamel. The mass of composite susceptible to be detected by the system will be quite lower than the 30 mg used in this study. Depending on the size and geometry of the restoration, only a few mg will be present within the sensitive volume of the resonator.

Based on the results obtained in X-band, we extended the study using a clinical L-band spectrometer developed by the Dartmouth EPR Center for in the field measurements, and a resonator specifically designed for the detection of the dosimetric signal of the enamel. One tooth was used for the whole study, in order to standardize as much as possible the setting, so that confounding factors were reduced to the minimum. The geometry of the tooth and of the composite tested were kept constant throughout the study. Under these conditions, only the influence of the composite should be measured, all other parameters being controlled and unchanged.

Using this more realistic setting, only one irradiated composite (IPS Empress Direct) showed a significant contribution to the dosimetric signal, with an overestimation about 30%. At lower doses, this overestimation is expected to be even higher. This overestimation could also be eliminated using an adequate fitting model. Indeed the radiation-induced and nitroxide-like signals from composites are different from the dosimetric signal of teeth in terms of shape and position (Fig 8), so that each contribution could be quantified separately after appropriate fitting.

From the operation standpoint, this overestimation should be acceptable, and should not fundamentally alter the decision making process for the appropriate management of overexposed individuals. More accurate dosimetry can be performed after triage, on a smaller number of subjects, when necessary. Nevertheless, only one geometry was considered in this study, so that the effect of the loss of enamel, due to the restoration, on the dosimetric signal was not completely investigated. Other geometries of restoration, including a variation of both the location of the restoration and its size, should be further investigated in order to complete the picture.

Another aspect possibly affecting the results is the storage conditions of samples (tooth and composites). In this study, all the samples were kept in dry conditions, but other storage conditions could change the situation to some extent. Teeth and restored teeth are naturally placed in a very humid atmosphere inside the mouth. This environment can affect both the kinetics of radicals induction/recombination and their detection, as EPR uses microwaves which are readily absorbed by water. Also, composites were polymerized out of the tooth, whereas in normal conditions they are cured directly in the tooth. Specific radicals due to the interaction between tooth tissue and restorative material cannot be excluded.

In conclusion, the influence of a radiation-induced EPR signal arising from the composites used for teeth restoration is rare and of low intensity. Nevertheless, the presence of a restoration will affect the mass of enamel detected, which is a more important parameter to take into account. A correction of the dose based on the loss of enamel material could be envisaged, using simple and fast imaging techniques (UV, IR or near IR) susceptible to detect and quantify the amount of restorative material, or reciprocally the loss of enamel material.

## Supporting Information

S1 Fig Spectrum of Gradia Direct X (A, B) and Artiste Nano (C, D) before (left) and after a 10 Gy irradiation (right). (TIF)
