# Supplementary figures and images for: Tooth Retrospective Dosimetry Using Electron Paramagnetic Resonance: Influence of Irradiated Dental Composites
Source: PLoS One. 2015 Jun 30;10(6):e0131913. doi: 10.1371/journal.pone.0131913 (PMC4488324; doi:10.1371/journal.pone.0131913)

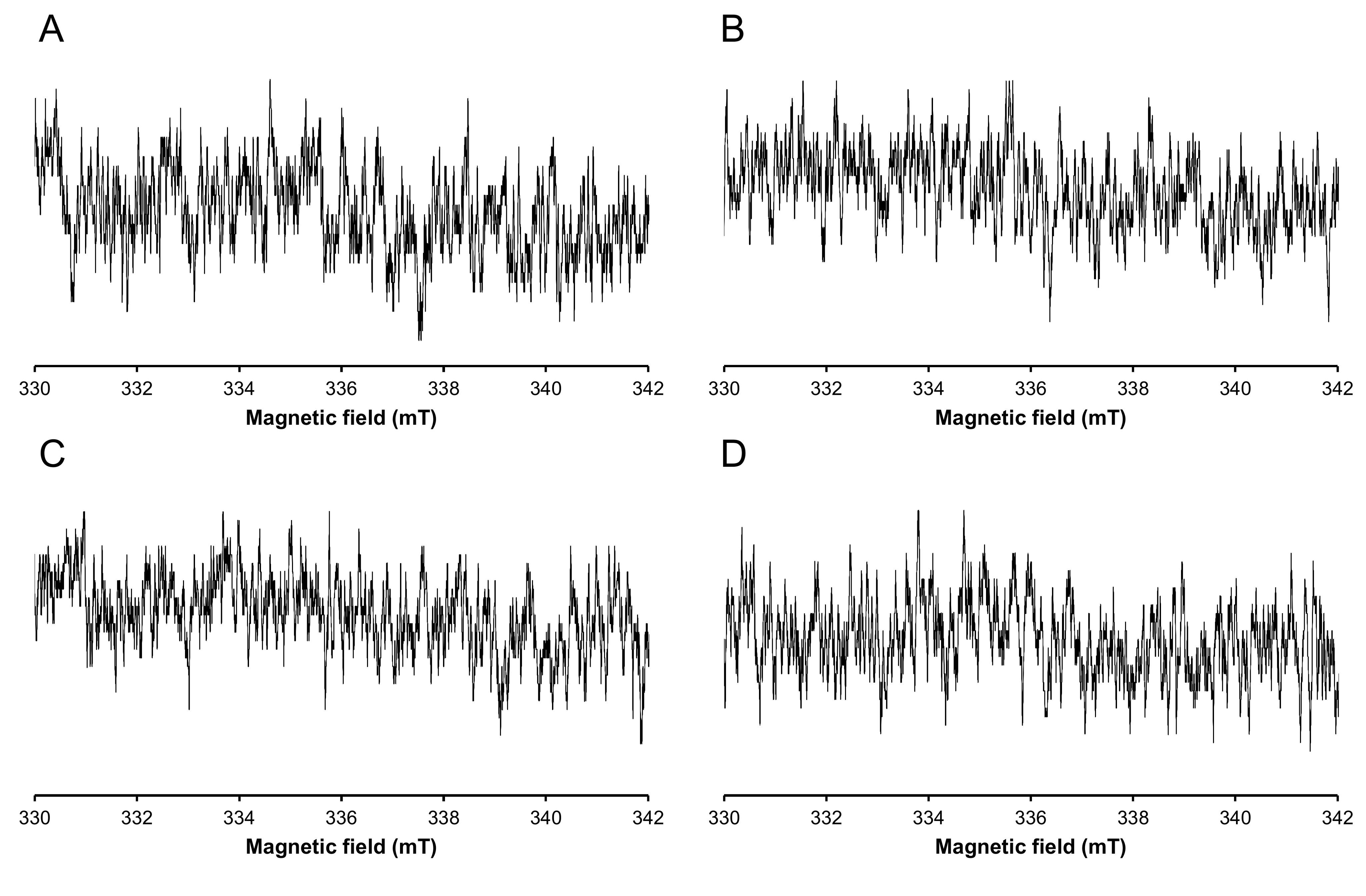

Supplement: S1 Fig — (TIF) [file pone.0131913.s001.tif]
